# Single-tunnel anatomic double-bundle anterior cruciate ligament reconstruction has the same effectiveness as double femoral, double tibial tunnel: A prospective randomized study

**DOI:** 10.1097/MD.0000000000014851

**Published:** 2019-03-15

**Authors:** Xianxiang Xiang, Zhenan Qu, Honglin Sun, Xiaojun Ma, Weiming Wang, Lixin Huang

**Affiliations:** a Department of Orthopedics, The First Affiliated Hospital of Soochow University, Suzhou, Jiangsu; b Department of Orthopedics; c Department of Nursing, The Affiliated Zhongshan Hospital of Dalian University, Dalian, Liaoning, China.

**Keywords:** anatomic reconstruction, anterior cruciate ligament, single femoral-single tibial tunnel

## Abstract

**Purpose:**

To investigate whether single femoral, single tibial tunnel anatomic double-bundle anterior cruciate ligament (ACL) reconstruction is equal to or superior to double femoral, double tibial tunnel ACL double-bundle anatomic reconstruction in terms of restoring the stability and functions of the knee joint.

**Methods:**

A prospective clinical study was performed to compare 30 cases of single-tunnel ACL double-bundle anatomic reconstruction to 28 cases of double-tunnel ACL double-bundle anatomic reconstruction, with average follow-up of 36 months. All graft tendons were hamstring tendon autografts. Tunnel placements in all the cases were made anatomically. Clinical results were collected after reconstruction. Graft appearance, meniscus status and cartilage state under arthroscopy were compared and analyzed.

**Results:**

Tunnel placements were in the anatomic positions in both groups. On the lateral pivot-shift test performed at 36 months postoperatively, there was no significant difference between groups. Clinical results such as International Knee Documentation Committee score, Tegner activity scale, and range of motion showed no significant differences between the groups. The mean thickness of anteromedial graft was reduced by 10.3% and that of the posterolateral graft was reduced by 11.1% from the original graft thickness evaluated by magnetic resonance imaging. No new meniscal tears were found either group; however, cartilage damage occurred in the double-tunnel group at 39.3%, and this rate was significantly higher than that in the single-tunnel group (10.0%).

**Conclusion:**

Single femoral, single tibial tunnel anatomic double-bundle ACL reconstruction has the same effectiveness as the double femoral, double tibial tunnel in restoring the knee's stability and functions.

## 1 Introduction

The anterior cruciate ligament (ACL) consists of 2 functional bundles, and each of the 2 primary ACL bundles has a unique function.^[1–3]^ The anteromedial (AM) bundle and posterolateral (PL) bundle are oriented near parallel with the knee extended, and twist around each other as the knee flexes.^[4]^ The AM bundle of the ACL is normally tighter in flexion and the PL bundle is tighter in extension.^[3]^ These bundles have different tension levels as the knee flexion angle changes and the PL bundle is particularly important to provide transverse plane rotational knee stability as the knee draws near full extension.^[1,5,6,7]^

On the basis of biomechanical studies and clinical trials, a double-bundle reconstruction technique has recently been proposed to better restore the anatomy and biomechanics of the native ligament.^[8]^ Biomechanical studies have found that anatomic double-bundle ACL reconstruction can restore knee stability significantly more closely to the normal level than conventional single-bundle reconstruction.^[9,10]^ In the early 2000s, some authors^[11,12]^ reported a new concept of anatomic reconstruction of the AM and PL bundles of the ACL with 2-year clinical results superior to those of conventional single-bundle ACL reconstructions. Some authors have reported that an anatomical ACL reconstruction can be achieved by reconstructing both the AM and PL bundles of the ACL using 2-femoral and 2-tibial tunnels.^[13–17]^ In this procedure, 2 femoral and 2 tibial bone tunnels are drilled at the anatomic ligament insertions. Other authors described an anatomic double-bundle ACL reconstruction technique with 1 femoral and one tibial tunnel and obtained good results.^[18–23]^ Their technique emphasized accurate tunnel placement within the femoral and tibial footprints as well as proper orientation of the bundles based on current anatomic knowledge. However, no patient outcome data were reported. Hemanth et al^[24]^ reported a fresh-frozen human cadaveric study with 9 samples and concluded that single-tunnel double-bundle ACL reconstruction better restored the anterior knee stability compared with conventional single-bundle reconstruction.

Nonsymptomatic tunnel communication seen on magnetic resonance imaging (MRI) has been reported in 10% to 19% of patients in the femur and in 24% to 29% of patients in the tibia on 1-to-2-year follow-up.^[25–27]^ Furthermore, some authors have proposed anatomical ACL reconstruction techniques using a single femoral and tibial tunnel as opposed to creating multiple tunnels.^[19,20,28,29]^ Nevertheless, the single-tunnel procedure is easier for surgeons and less traumatic for patients compared to double-tunnel, few studies have investigated the efficacy of such ACL reconstruction techniques. Therefore, in this study, we hypothesized that tibia-femoral single tunnel double-bundle ACL reconstruction would produce the same effect in terms of restoration of anterior and rotational stability, and would provide the same or better objective clinical results, as would tibia-femoral double-tunnel reconstruction.

## 2 Materials and methods

### 2.1 Patients

From April 2011 to April 2013, 263 patients with ACL injury underwent ACL reconstruction in Affiliated Zhongshan Hospital of Dalian University and The First Affiliated Hospital of Soochow University. Because the research has been focused on isolated ACL injury only, the inclusion criteria for the choice of patients were as follows: non-professional athletes and non-heavy manual workers; less than 6 months after injury; the other knee healthy; and younger adults aged 18 to 40 years. Injuries combined with posterior cruciate ligament injury, osteoarthritis, meniscal injury, MCL or PL corner injuries were excluded. All surgeries were performed by 2 experienced senior surgeons. Ethics approval was obtained from the Institutional Ethics Committee of Dalian University. Patients who fulfilled the inclusion criteria were randomized into 2 groups by means of a computer-generated list of random numbers. Finally, 58 patients underwent ACL reconstruction with either tibia-femoral single-tunnel or tibia-femoral double-tunnel procedures: single-tunnel group (ST group; n = 30) and double-tunnel group (DT group; n = 28). All graft tendons were hamstring tendon (HT) autografts, and the hamstring tendon was harvested from the patient's normal-functioning limb on the opposite side of injury because of the possibility of tendon extraction affecting rotational instability. All patients underwent preoperative examinations, including Lachman testing, anterior drawer testing, and pivot-shift testing. They were also tested with a KT-1000 arthrometer with knee flexion of 30° and 90° at 134 N and manual maximum force (MEDmetric, SanDiego, CA). All patients were evaluated with International Knee Documentation Committee (IKDC) subjective scores, Lysholm scores, and Tegner scores and were instructed to return for follow-up arthroscopic evaluations. By the end of the study, all 30 subjects in the ST group and 28 subjects in the DT group had followed up for 30 months. The demographic data of the 2 groups are displayed in Table 1.

**Table 1:**
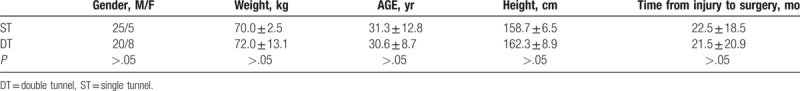
Demographic data of the 2 groups.

#### 2.1.1 ST ACL reconstruction

**Figure 1:**
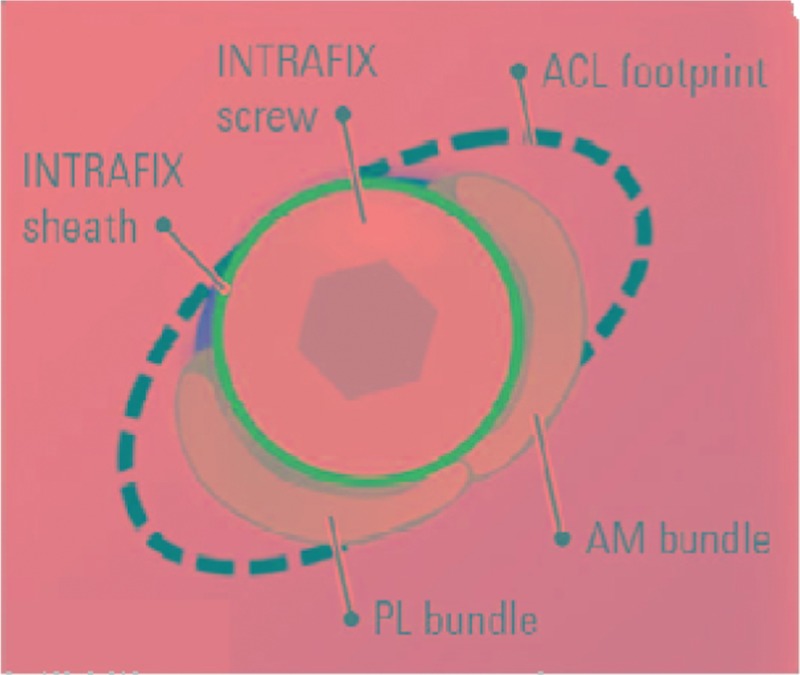
This is the model of the ACL footprint on femoral. ACL = anterior cruciate ligament.

**Figure 2:**
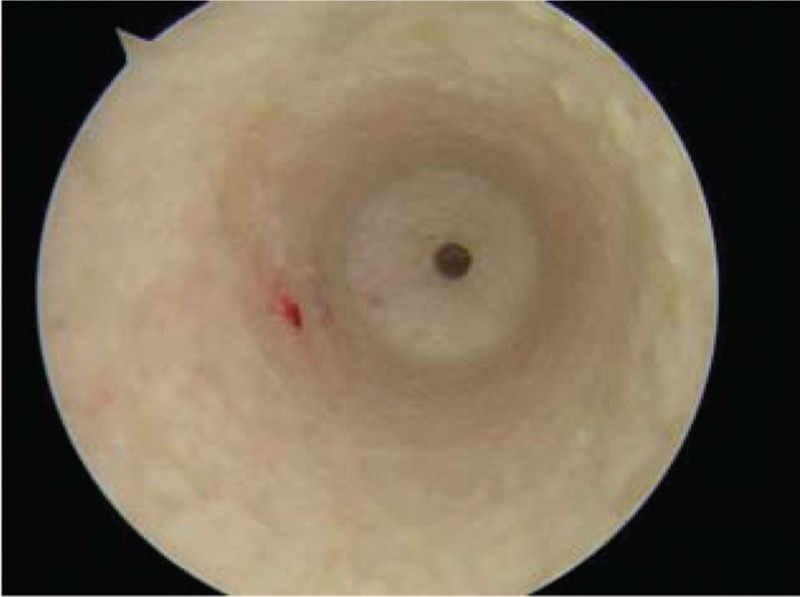
This is the femoral tunnel, the intactness bonny wall.

**Figure 3:**
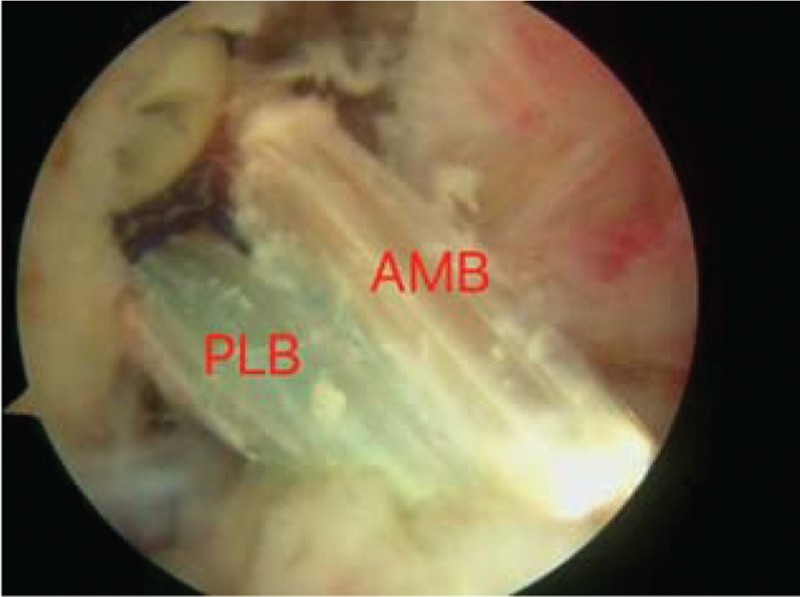
The AM bundle and PL bundle are near parallel with the knee extended. AM = anteromedial, PL = posterolateral.

**Figure 4:**
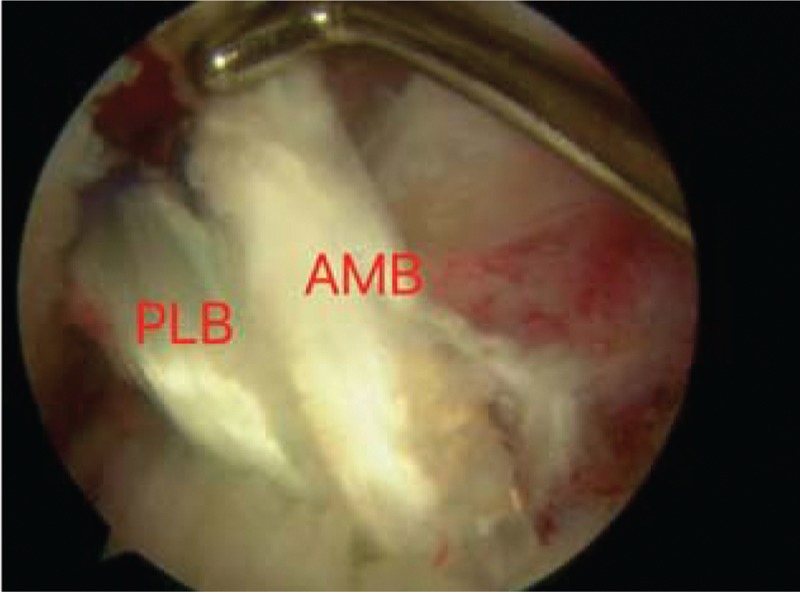
The AM bundle and PL bundle twist around each other as the knee flexes. AM = anteromedial, PL = posterolateral.

**Figure 5:**
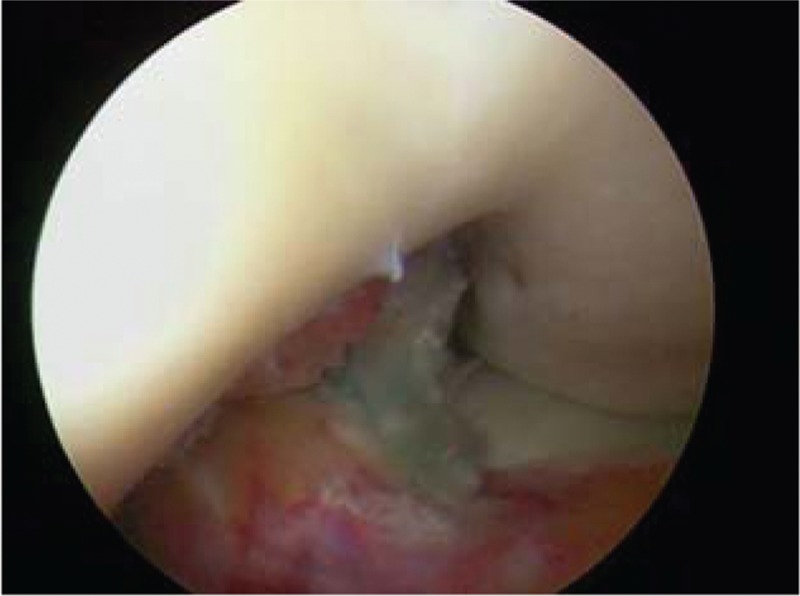
When the knee extended, no impingement occurred.

When ruptured ACL was verified under arthroscopic visualization, the arthroscope was used in the knee through the anterolateral portal. The tibial tunnel was placed at the center of footprint of the ACL with the tibial tunnel guider at 90° of the knee, and the tunnel at 45° to the tibia shaft. Both the lateral intercondylar ridge and the lateral bifurcate ridge are important bony landmarks for the femoral attachments of the ACL, and the femoral tunnel was placed in the center of the lateral bifurcate ridge but did not surpass the lateral intercondylar ridge. It was drilled through the AM portal with a cannulated reamer using a freehand technique at 120° of the knee. Both tibia and femoral drills were selected according to the graft diameter. Then, the knee was relaxed to allow the lower leg to droop in a natural position. The surgeon takes the HT autograft into the femoral tunnel through the tibia tunnel, making sure that the 2 bundles were arranged from AM to PL in the femoral tunnel, then drives into a sheath that isolates the 2 bundles, and screws the bio-absorbable interference screw in the sheath through the AM portal, keeping the knee at an angle of 120°. The grafts were fixed with bioabsorbable interference screws using an outside-in technique in the tibia and an inside-out technique in the femur (Figs. 1–5).

#### 2.1.2 DT ACL reconstruction

As in the ST procedure, rupture of the ACL under arthroscopy was verified. The tibial AM tunnel was located in the anterior part of the footprint and the tibial PL tunnel was in the posterior part of the footprint. The femoral tunnels were created via an AM portal with a freehand technique for the anatomic insertions of the AM and PL bundles of the ACL at 120° of the knee. The bony wall between the femoral tunnel apertures was at least 1 to 2 mm. The PL graft was first taken through the PL tunnel and the AM graft was taken later. The grafts were fixed with bioabsorbable interference screws using an outside-in technique in the tibia and an inside-out technique in the femur. The other procedures were the same as the ST ACL procedure. Neither impingement nor cartilage damage was observed under arthroscopy in all patients.

#### 2.1.3 Rehabilitation

All knees were braced and the brace was not taken off until 8 weeks. The knee should be kept in full extension during the first 2 weeks. Both the ST and DT group received the same standard post-operative rehabilitation program, with full weight-bearing allowed at least 4 weeks after surgery, and full range of motion (ROM) was obtained within 8 weeks. Running was allowed only at 4 months; however, contact sports were not recommended until 8 months after the operation.

#### 2.1.4 Evaluation of graft findings in MRI

**Figure 6:**
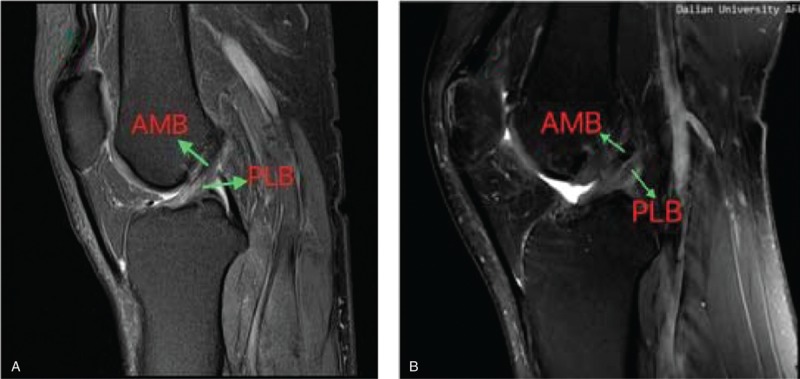
a: on oblique sagittal MRI, the 2 bundles were seen clearly, and near parallel with the knee extended (Healthy people);b: Under oblique sagittal MRI, the 2 bundles were seen clearly, and near parallel with the knee extended (postoperation). MRI = magnetic resonance imaging.

**Figure 7:**
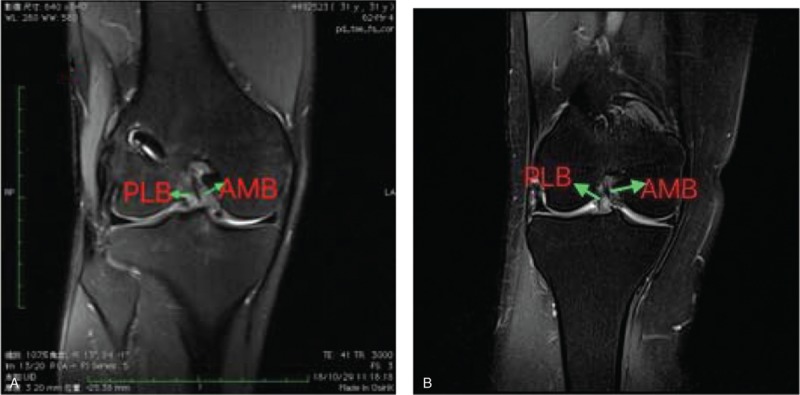
On oblique coronal MRI, the 2 bundles were seen clearly. a:postoperation; b: healthy people. MRI = magnetic resonance imaging.

**Figure 8:**
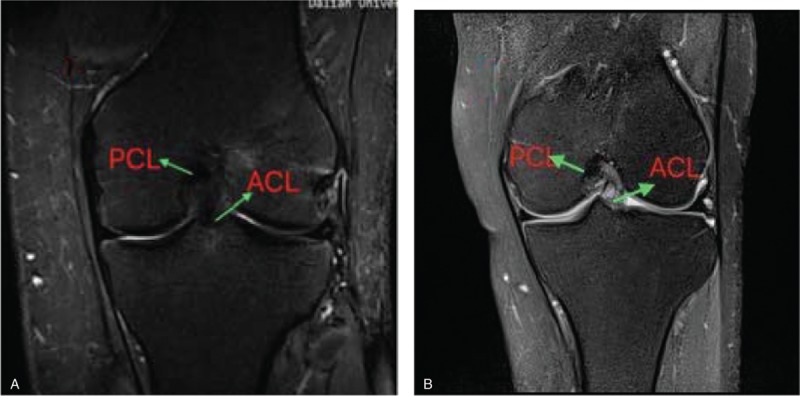
a; the foofprint of tibial postoperation of ACL-R; b: ACL footprint of helthy people. ACL = anterior cruciate ligament.

**Figure 9:**
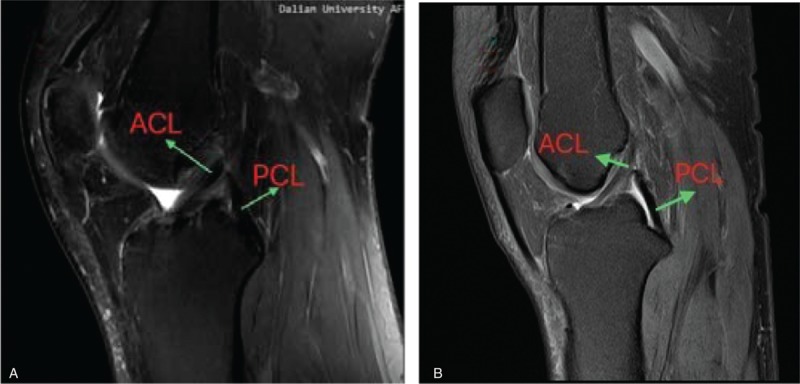
a: the ACL shape on MRI in patients postoperation; b: the ACL shape on MRI in healthy people. ACL = anterior cruciate ligament, MRI = magnetic resonance imaging.

**Figure 10:**
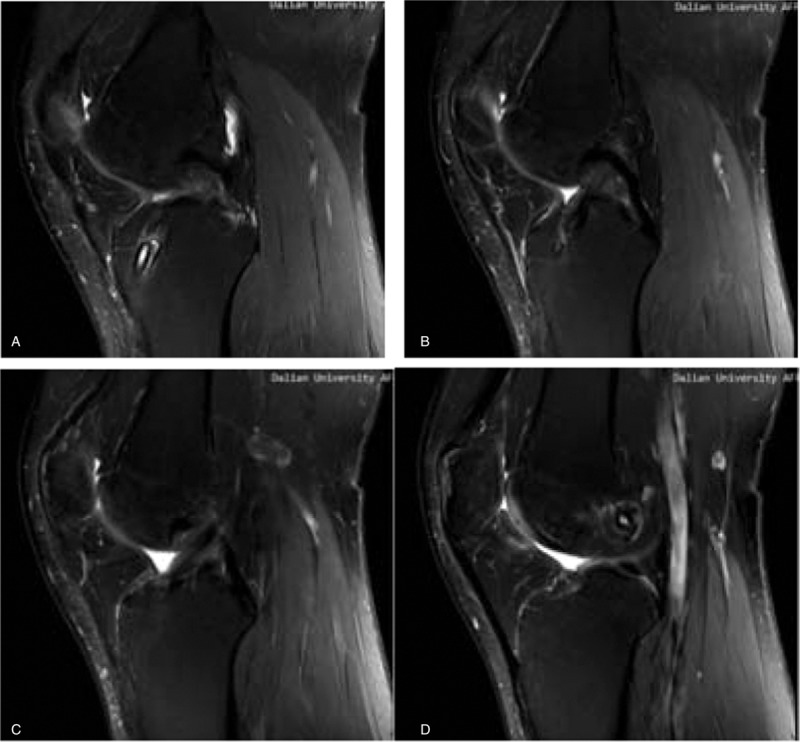
On oblique sagittal MRI. MRI = magnetic resonance imaging.

**Figure 11:**
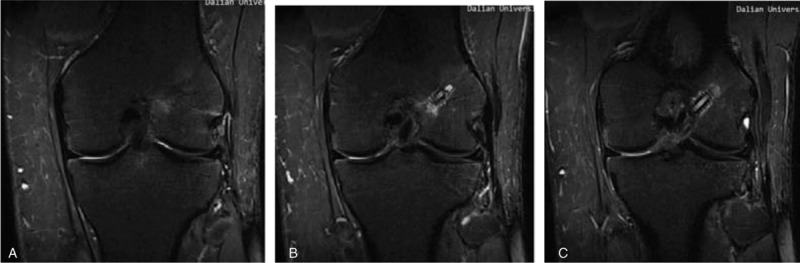
On oblique coronal MRI. MRI = magnetic resonance imaging.

MRI was carried out at a mean 26 months (range 24–30 months) postoperatively with a 3.0-T Sigma Excite HD imager (GE Medical Systems, Milwaukee, WI) using an 8-channel receiver/transmitter extremity coil. The imaging planes were chosen the same way in all patients. The knee was placed into the coil in a slight flexion of a mean 8° resulting from the shape of the coil and imaged at rest (Figs. 6–11).

#### 2.1.5 Clinical effectiveness follow-up

All patients were examined at 1, 3, 6, 12, 18, 24, and 36 months after surgery. At the final visit, an evaluation was made and the appearance of the grafts, the meniscus, and the cartilage were observed and compared with those before surgery under arthroscopy. The classification system described by Kondo and Yasuda^[30]^ was used to evaluate the grafts: 4 points, excellent; 2 or 3 points, fair; and 0 or 1 point, poor. Clinical outcomes were assessed by ROM, Tegner score, pivot-shift test, joint laxity testing as evaluated with the KT1000 (Med Metric Inc. TX), Lachman test and IKDC subjective score.

#### 2.1.6 Statistical analysis

Statistical analysis comparing 2 groups were conducted using the independent samples *t* tests, and the chi-square test. SPSS statistical software (version 24.0; Chicago, IL) was used for statistical calculations. *P* value <.05 was used as the level of significance.

## 3 Results

All patients were followed up for 36 months. No complications due to early graft failure or superficial or deep infections were reported in either group. At the latest follow-up, no re-ruptures were observed in any subject.

In the current health assessment section, there were significant differences between the preoperative and postoperative evaluations for each group; however, no significant differences were found in either group after operation.

Thigh muscle strength evaluation was performed for both groups. The percentage of isokinetic peak torque of knee flexion and extension for the operated knee compared to the contralateral normal knee was 90% ± 16 and 95% ± 21, with no patient in either group showing positive instability. The mean thickness of the original graft (femoral drill size) in the operation was 6.4 mm (range 6.0–7.5 mm) for the AM graft and 5.9 mm (range 4.0–6.0 mm) for the PL graft in the DT group, while the thickness of original graft was 6.2 mm (range 4.9–6.5 mm) in the ST group both AM and PL). On MRI, the mean AM graft AP (anteroposterior) thickness was 5.8 mm (range 4.0–8.0 mm) in the ST group and 5.9 mm (range 4.0–9.0 mm) in the DT group, while the mean PL graft AP thickness was 5.5 mm (range 3.9–7.0 mm) in the ST group and 5.1 mm (range 4.0–8.0 mm) in the DT group. Compared with the mean original graft thickness, the AM graft was reduced by 10.3% and the PL graft by 11.1% in diameter (Table 2). The interobserver agreement on measurements of the graft thickness between the 2 radiologists was excellent.

**Table 2:**
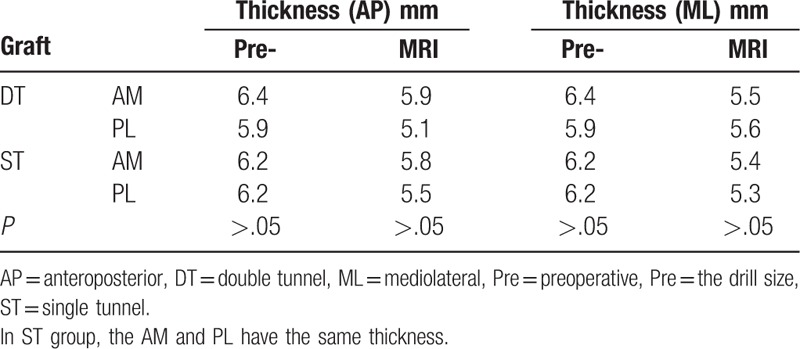
The graft evaluation.

There were no new concomitant meniscus injuries in either group. Cases with cartilage lesions were counted and listed in Table 3, and the cartilage lesions were more severe than grade II in various parts of the knee joint before and after the reconstruction. There were 3 cases (10.0%) and 11 cases (39.3%) of new cartilage lesions in the ST group and DT group, respectively, significantly more in the DT group (*P* <.05). The increased lesions were in the medial femoral condyle, trochlea, and patella. In addition, the rate of cartilage injury incurred during the operation in the DT group was significantly higher than that of the ST group.

**Table 3:**
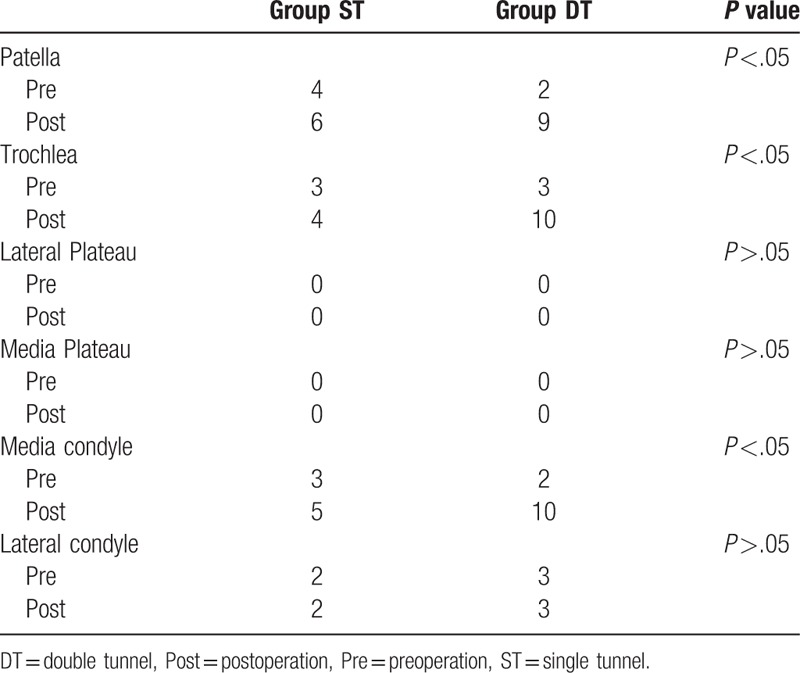
Cartilage status.

The clinical results of last follow-up are listed in Tables 4 and 5. There were no significant differences between the groups. Both methods can restore the function of the knee.

**Table 4:**
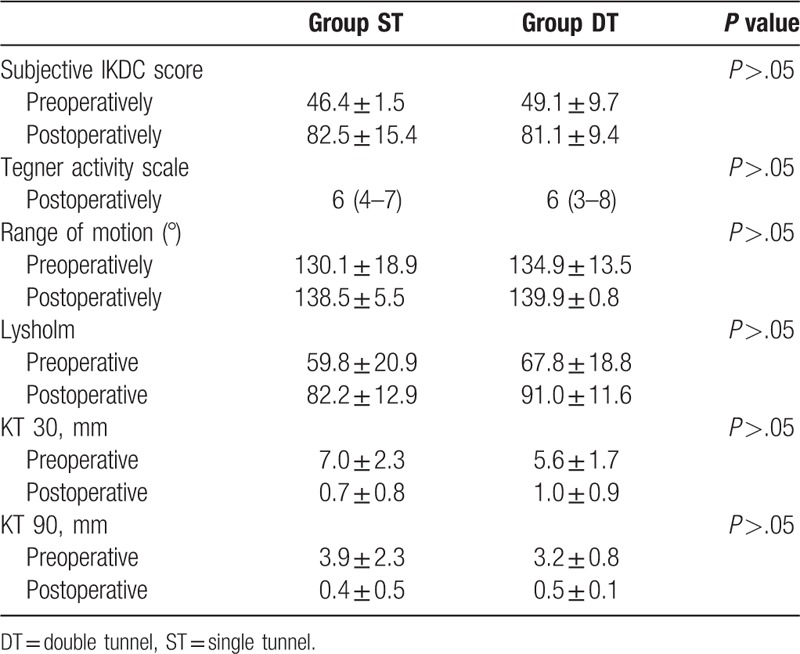
Clinical results of the ST and DT group at months after surgery.

**Table 5:**
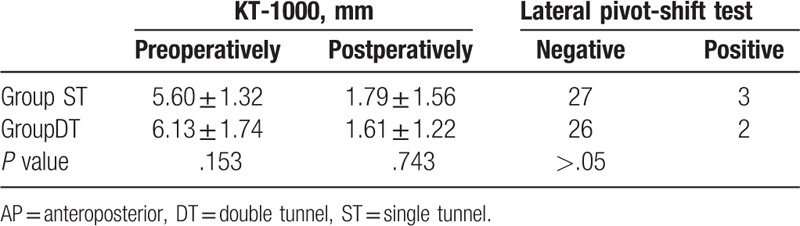
Evaluation of AP and rotational instability at 36 months after surgery in both groups.

## 4 Discussion

For a more successful reconstruction of the ACL, the ideal outcome would be restoration of the original function of the ACL. In recent years, the number of anatomic double-bundle ACL reconstruction (DB-ACLR) has increased rapidly. Laboratory and clinical studies demonstrated that the procedure resulted in better rotational stability than was afforded by traditional SB (single-bundle) ACL reconstruction, with fewer graft failures,^[4,31–34]^ as shown in a review of 14 randomized controlled trials by Järvelä and Suomalainen.^[35]^ A meta-analysis performed by Yunes et al^[36]^ estimated that only 60% to 64% of patients undergoing single-bundle ACL reconstruction returned to their preinjury levels of activity. Kidera et al^[37]^ used a 2D/3D registration technique and found that DB-ACLR improved not only tibial AP instability but also tibial rotational instability. DB-ACLR appears to be a useful technique for normalizing the knee joint kinematics of ACL deficient knees. Intraoperative measurement studies showed that the clinically available anatomic double-bundle procedures can reconstruct knee stability significantly better and can improve knee function close to the normal level at the time immediately after surgery compared with the conventional single-bundle procedure.

The location of the tunnels was of critical importance to the anatomic reconstruction of the ACL. To anatomically reconstruct the ACL, the placement of the femoral or tibia tunnel should be within the original insertion site area. The majority of the studies on ACL reconstruction reported 80% to 90% patient outcome success rates; however, approximately 10% to 30% of patients continue to experience persistent knee pain or instability.^[38,39]^ If revision surgery is necessary, the most prevalent cause is faulty surgical technique, particularly improper tibia and femoral bone tunnel placement.^[40–42]^ Therefore, when we decide to perform anatomic double-bundle ACL reconstruction, we should choose a method with fewer complications.

In our procedure, a critical point in drilling the femoral tunnels is the position of the AM portal. It should be approximately at the level of the ACL footprint, not too medial; otherwise, the drill bit will injure the articular surface of the medial femoral condyle and the exit of the tunnel will be too lateral on the posterior femoral notch, increasing the used length of the graft. If it is too far lateral, the femoral tunnels will be oblique with oblong orifices and the posterior cortex will be thin and weak, with the risk of blowout. Giron et al^[43]^ compared femoral tunnel position using double incision, transtibial, and AM portal tunnel drilling techniques and reported that each could be used to effectively achieve sufficiently deep femoral tunnel positioning. Cadaveric studies showed that better rotatory stability control was achieved when the graft was placed with the femoral tunnel in a more horizontal position.^[12]^ Therefore, in this study, we chose the AM portal, because in this way, the tunnel would be more horizontal than in the others, and this approximates the anatomy. One advantage of drilling a femoral tunnel through an accessory portal is the ability to choose the optimal position of the tunnel, without needing to follow the direction set by the tibia tunnel.

In this study, autogenous HT grafts were chosen in the anatomic anterior cruciate ligament reconstruction (ACL-R). Using the HT graft for double-bundle reconstruction of the ACL is becoming more popular with time. Ertoğrul et al^[44]^ found that there were no differences, clinically or functionally, between the autograft and allograft groups at final follow-up (*P* >.05). In another study, Brian et al^[45]^ compared the hamstring autograft and patellar tendon autograft and concluded that both graft types remain viable options for primary ACL reconstruction. Patients undergoing ACL reconstruction with bone-patellar tendon–bone (BTB) and HT grafts show comparable improvement in functional results after 1 year of rehabilitation.^[46]^ Cory et al^[47]^ suggested there were no differences in laxity and that clinical outcome scores 3 to 6 years after surgery were similar to those with allograft tendons with autografts. A study of the graft choice for ACL-R concluded that patients undergoing anatomic ACL-R should ideally receive an autograft, even if it requires harvesting of the HT autograft from the contralateral knee.^[48]^

In this single-tunnel double-bundle ACL reconstruction technique, the 2 bundles in the joint are positioned in a more anatomic fashion, improving the kinematic performance of the grafts with promising better results, especially in terms of rotation control. There were no significant differences between ST and DT group either in clinical outcomes or in anterior-posterior and rotational stabilities in our study. Similar results were observed in another study that evaluated the efficacy of a single-tunnel double-bundle ACL reconstruction technique in restoring normal knee kinematics.^[20,28,29]^ Hemanth et al^[24]^ performed a study on 9 fresh-frozen human cadavers and concluded that single-tunnel double-bundle ACL reconstruction can better restore the anterior knee stability compared with conventional single-bundle reconstruction. Based on our data, the normal anterior stability of the knee joint can be efficiently restored by an anatomical single-tunnel ACL reconstruction. Furthermore, the rate of cartilage injury in the DT group was significantly higher than that of the ST group. Such techniques could be easily adopted by surgeons who currently practice single-bundle ACL reconstruction to achieve a more anatomical reconstruction of the ACL.

Some studies demonstrated that ACL-R not only cannot fully prevent development of OA but, in certain occasions, ACL-R may be associated with a higher prevalence of knee OA. Specifically, a retrospective cohort study at 11 years post ACL injury showed that only 25% of conservatively treated knees developed OA versus 42% in ACL reconstructed knees^[49]^ One theory that aims to explain the increased incidence of OA in ACL injured knees without associated injuries is the theory of the initial impact. According to this theory, acute impact trauma to the articular cartilage initiates a degeneration process that can progress to osteoarthritis over the next years after the event. During ACL injury, pro-inflammatory cytokines, such as interleukin-6, interleukin-8, tumor necrosis factor-alpha, and keratan sulfate are increased and can remain elevated even 3 months after the injury.^[50,51]^ A cohort study with yearly follow-up assessment for 11 years showed that cartilage degradation as a result of the initial impact typically accelerates after 5 years from the injury.^[52]^ These recent data suggest that at the time of ACL injury a degradation process of the cartilaginous matrix initiates, either as the result of direct impact or due to alterations in joint homeostasis after the impact injury to the joint. Therefore, in our study, we think the operation time and the more tunnels maybe contribute to the higher rate of cartilage injury in DT group, the operation is the second trauma for the knee after all.

MRI is the preferred imaging modality for the evaluation of ACL graft reconstructions.^[53]^ According to comparing the diameter of the grafts before and post-operation, we can find partial rupture or disruption of graft. For the clinical point of view, the disruption of grafts is an important indication for the revision ACL surgery if the patient complains of symptoms of instability in the operated knee as well.

The surgical procedure in ST group was simpler than that in DT group; the young surgeons could master it easily. The learning curve of ST-ACLR was shorter than that of DT-ACLR. As the number of tunnels decreases, we can retain more bones and use fewer screws in surgery can reduce the financial burden of patients. We think these are the most important findings in this research, restore the knee function, reduce the economic burden, and more surgeon master this procedure.

There are some limitations in this study. First, we performed the pivot-shift test as the method to assess rotational stability and found no differences; however, in our experience, it is a subjective test that is prone to inter-examiner variation. Therefore, we need more objective clinical tests to reflect the true functions of the knee. Second, longer-term follow-up is essential to explore the biologic behavior of the tendon-bone healing of the graft. Third, in spite of the good results we obtained, and the small number of patients in this study, we need more clinical practice to support it.

## 5 Conclusions

Patients with the anatomic ST-DB-ACL reconstruction can obtain clinical outcomes as satisfactory as those of the DT groups. Although there remains much to learn about anatomic ACL reconstruction methods, further studies are required to confirm the clinical role of ST-DB-ACLR. Furthermore, our results indicated that ST-DB-ACLR could be 1 more surgery choice for the patients who suffer from ACL rupture.

## Author contributions

**Conceptualization:** Zhenan Qu, Xiaojun Ma, Lixin Huang.

**Data curation:** Zhenan Qu, Xiaojun Ma.

**Formal analysis:** Xianxiang Xiang, Zhenan Qu.

**Methodology:** Lixin Huang.

**Project administration:** Lixin Huang.

**Resources:** Honglin Sun.

**Software:** Honglin Sun.

**Supervision:** Honglin Sun, Weiming Wang.

**Visualization:** Weiming Wang.

**Writing – original draft:** Xianxiang Xiang, Zhenan Qu.

**Writing – review & editing:** Xianxiang Xiang.
